# Prevalence and associated factors of depressive and anxiety symptoms during pregnancy: A population based study in rural Bangladesh

**DOI:** 10.1186/1472-6874-11-22

**Published:** 2011-06-02

**Authors:** Hashima E Nasreen, Zarina N Kabir, Yvonne Forsell, Maigun Edhborg

**Affiliations:** 1 Research and Evaluation Division, BRAC, 75 Mohakhali, Dhaka 1212, Bangladesh; 2 School of Public Health, BRAC, 66 Mohakhali, Dhaka 1212, Bangladesh; 3 Department of Neurobiology, Care Sciences and Society, Karolinska Institutet, Sweden; 4 Department of Public Health Sciences, Public Health Epidemiology, Karolinska Institutet, Sweden

## Abstract

**Background:**

Few studies have examined the associated factors of antepartum depressive and anxiety symptoms (ADS and AAS) in low-income countries, yet the World Health Organization identifies depressive disorders as the second leading cause of global disease burden by 2020. There is a paucity of research on mental disorders and their predictors among pregnant women in Bangladesh. This study aims to estimate the prevalence of depressive and anxiety symptoms and explore the associated factors in a cross-section of rural Bangladeshi pregnant women.

**Methods:**

The study used cross-sectional data originating from a rural community-based prospective cohort study of 720 randomly selected women in their third trimester of pregnancy from a district of Bangladesh. The validated Bangla version of the Edinburgh Postnatal Depression Scale was used to measure ADS, and a trait anxiety inventory to assess general anxiety symptoms. Background information was collected using a structured questionnaire at the respondents' homes.

**Results:**

Prevalence of ADS was 18% and AAS 29%. Women's literacy (OR 0.59, 95% CI 0.37-0.95), poor partner relationship (OR 2.23, 95% CI 3.37-3.62), forced sex (OR 1.95, 95% CI 1.01-3.75), physical violence by spouse (OR 1.69, 95% CI 1.02-2.80), and previous depression (OR 4.62 95% CI 2.72-7.85) were found to be associated with ADS. The associated factors of AAS were illiteracy, poor household economy, lack of practical support, physical partner violence, violence during pregnancy, and interaction between poor household economy and poor partner relationship.

**Conclusion:**

Depressive and anxiety symptoms are found to occur commonly during pregnancy in Bangladesh, drawing attention to a need to screen for depression and anxiety during antenatal care. Policies aimed at encouraging practical support during pregnancy, reducing gender-based violence, supporting women with poor partner relationships, and identifying previous depression may ameliorate the potentially harmful consequences of antepartum depression and anxiety for the women and their family, particularly children.

## Background

Historically, pregnancy is a time of enjoyment and fulfillment for women. However, evidence indicates that there is an increase in psychiatric morbidity, particularly depression and anxiety, during this period [1]. Depressive symptoms during pregnancy may have devastating consequences, not only for the women, but also for the child and family [2]. Antepartum depressive and anxiety symptoms (ADS and AAS, respectively) can lead to postpartum depression [3,4], lower birth weight, and premature delivery [5], and have a negative impact on child development [6,7].

A meta-analysis of 21 studies reported the mean prevalence of ADS to be 12% in high-income countries [8]. Yonkers et al. reported the prevalence of AAS to be 24% [9]. Studies in high-income countries suggest a link between disadvantaged socioeconomic background, domestic violence, and ADS and AAS [5,8].

Despite World Health Organization (WHO) estimates that depressive disorders will be the second leading cause of global disease burden by 2020 [10], only a few studies on ADS and even less on AAS have been conducted in low-income countries. Two community-based studies of pregnant women in Pakistan found that 18% were anxious and depressed [11] and 25% were depressed [12]. The prevalence of depression during the third trimester was about 16% among South Indian women [13]. The predictors of antepartum depression and anxiety in Pakistan were husband's unemployment, low household wealth, 10 or more years of formal education, unwanted pregnancy, and partner violence [11]. In a study in Nigeria, socioeconomic and obstetric factors were not found to be associated with ADS and AAS [14]. Age at current pregnancy and at first delivery, obstetric complications, having no friends in the community, living in a crowded household, lower occupational status, and history of previous psychiatric disorder were found to be associated with common mental disorders in Brazil [15]. Depression and anxiety during pregnancy are rarely reported in Bangladesh. One study involving 316 pregnant women in a rural subdistrict in the southwest part of Bangladesh found a 33% prevalence of ADS [16]. The study reported that partner violence, unsupportive husband and/or mother-in-law, and family preference for son were predictors of antepartum depression. The few studies and partly inconsistent results emphasize the need for further research on antepartum depression, particularly antepartum anxiety among women in low-income countries. This study aims to examine and identify the prevalence of potential contributors to antepartum anxiety and depressive symptoms among women in a rural area of Bangladesh.

## Methods

### Study setting

The data used in the study originated from the cross-sectional first phase of a prospective longitudinal study of perinatal depressive and anxiety symptoms among women in rural areas of two subdistricts of the Mymensingh district (120 km north of the capital city of Dhaka) of Bangladesh. As is typical of rural Bangladesh, the economy in the study area is agrarian, and approximately 40% of the population lives below the poverty line. The majority of women are involved in household work and child care. A national nongovernmental development organization, BRAC (formerly the Bangladesh Rural Advancement Committee), provides a variety of services in the area for the social and economic development of communities. The BRAC health program provides education in preventive health and nutrition, as well as immunization, family planning, pregnancy and reproductive health care, and basic curative services. The community health volunteers of BRAC identify pregnancies during the first trimester, estimate gestational age (based on the last menstrual period reported by the women), confirm pregnancies at 4-5 months, and register them.

### Sample

The pregnancy registration maintained by the BRAC health program provided the sampling frame for this study. The gestational age recorded in the register was verified by the interviewers during data collection. The mandatory requirements for participation were residence in the area and being at third trimester of pregnancy; in other words, the length of pregnancy was 7 months and on. The exclusion criteria for the original study were emigration from the study area, intrauterine death, and mother's and child's death. Twenty-five selected pregnant women were not found to be available at their homesteads in three consecutive visits and were then replaced by the pregnant women in the next household. There is no significant difference in background characteristics between the studied women and the women who were not available. A cohort of 720 consecutive women was studied from the third trimester of pregnancy to 6-8 months postpartum. With an average 1,250 population per village in Bangladesh and a delivery rate of 3%, 37 women were expected to give birth in each village/year. Therefore, 154 villages were needed to obtain the required sample. Assuming an estimated prevalence of depression of 20% in India [17] and Pakistan [18] (no prevalence figure was available for the Bangladeshi population when the study was initiated), the study was designed with a precision of 0.05, a power of 0.80, and an effect size of 0.40 to detect the difference between depressed and nondepressed women.

### Data collection

Data collection was performed from July 2008 to August 2009. Data at baseline (third trimester of pregnancy), including information on socioeconomic condition, reproductive health, family support, intimate partner violence, and anxiety and depressive symptoms, were collected through structured interviews by ten trained female interviewers at the respondents' homes. The interviewers were sociologists or anthropologists that had received two weeks training on the questionnaire (Additional file 1) and data collection procedure and did not have any formal linkage with the BRAC health services. They had resided in the local area for a period of one year and built a sympathetic and trustworthy relationship with the community. The questionnaire was composed, and interviews were carried out, in Bangla.

### Explanatory variables

#### Assessment of socioeconomic, obstetric, family support, and intimate partner violence

The age of each woman was calculated in years. Socioeconomic status was indicated by the woman's literacy (ability to read and write) and years of schooling, husband's literacy, and economic status of the household. Three measures were used to assess household economy: the woman's involvement in income-earning activities, the amount of land owned by the household, and per capita daily household expenditure on food. Obstetric indicators encompassed the numbers of live and dead children, planned current pregnancy, and antenatal consultation by health personnel. Family support was indicated by practical support from family members during pregnancy and the overall relationship with the husband and mother-in-law. Practical support included any kind of domestic help in household chores, such as cooking, washing, cleaning, or child rearing. Intimate partner violence encompassed physical violence ever, forced sex ever, and physical violence during pregnancy. Physical violence included being slapped, shoved, punched, kicked, or dragged on the ground by the partner [19]. Physical violence was scored from 0 to 4 based upon the number of violent acts faced. Forced sex was considered if the women reported that they had been forced by their husbands into sexual intercourse.

Based upon prior research [1,2,11,12,14,16], the explanatory variables were dichotomized, categorized, or used as a continuous scale. Maternal age was expressed as ≤20 years, 21-34 years, and ≥35 years; woman's literacy as yes or no; household economic status as poor or nonpoor [poor household economic status was indicated by a per capita daily household expenditure on food of less than the median of BDT 31.25 (USD 0.45)]; woman's involvement in income-generating activities as yes or no; experience of child death as yes or no; planned current pregnancy as yes or no; parity as primiparous or multiparous; practical support as support received or no support received during pregnancy; relationship with husband as poor or good; experienced physical violence as none or few acts (one or two) of violence versus multiple (three to four) acts of violence; forced sex ever as yes or no; and, previous history of depressive symptoms as yes or no.

### Outcome variables

#### Assessment of antepartum depressive symptoms (ADS)

The Edinburgh Postnatal Depression Scale (EPDS) [20] was used to detect depressive symptoms. The EPDS is a 10-item questionnaire, scored from 0 to 3 (higher score indicating more depressive symptoms), that has been validated for detection of depression in antepartum and postpartum samples in many countries [21]. The instrument was validated in Bangladesh for postpartum use (EPDS-B) and showed a sensitivity of 89% and specificity of 87% at the cutoff score 9/10 [22]. This cutoff score was used to categorize depressed (score ≥10) and nondepressed (score <10) states. The scale demonstrates good reliability in the present study with a Cronbach's alpha of 0.87 for assessment of ADS.

#### Assessment of antepartum anxiety symptoms (AAS)

General anxiety was assessed using the trait-anxiety scale of the State-Trait Anxiety Inventory (STAI), which was validated against the pregnancy-related anxiety questionnaire and is often used to assess anxiety in pregnancy [7,23]. STAI-trait consists of 20 items scoring from 1 to 4. The scale assesses anxiety levels in general, such as feelings of pleasure, nervousness, restlessness, satisfaction, or happiness. STAI-trait is a reliable and valid measure that can be used in both clinical and general populations [23]. A cutoff score of 45/46 was used to categorize anxious (score ≥45) or not-anxious (score <45) states [24]. Translation and back translation of the items on the scale was done by two bilingual social science researchers. Qualitative in-depth interviews with rural pregnant women outside the study area on the items of STAI helped to identify appropriate terminology in Bangla. STAI demonstrated good internal consistency in the present study with a Cronbach's alpha of 0.82 and split-half correlations of 0.81 for antenatal assessment.

### Analysis

Descriptive analyses were performed for the background characteristics of respondents and prevalence of ADS and AAS. An independent t-test was used to compare means between groups. Bivariate analyses (*X^2 ^*test and Fisher's exact two-sided p test) were conducted between each independent variable and the outcome variables ADS and AAS independently to identify possible contributory factors. The independent variables that were significantly associated (p < 0.05) with the outcome variables in the bivariate analysis were considered as possible contributory factors and entered into the regression models. We reported an odds ratio (OR) at a 95% confidence level to indicate the likelihood of reporting depressive and anxiety symptoms. We used the Enter method in a multiple logistic regression model to determine the associated factors of ADS and a multiple linear regression model for AAS. As EPDS was used with a definitive cutoff to indicate women with or without depressive symptoms, we used it as a dichotomized outcome variable in the multiple logistic regression model, and the independent variables were dichotomous or categorical. STAI-trait did not have any definitive cutoff and was used as a continuous scale in the multiple linear regression model. Independent variables such as age, per capita daily household expenditure on food, and acts of physical violence were used as continuous scales. Any violation of assumption was observed by examining the interaction between explanatory variables and outliers in both models, and diagnostic tests in the linear regression model.

### Ethical consideration

The study was approved by the Bangladesh Medical Research Council [BMRC/Eth.C/2008/402] in Bangladesh and the regional board for research ethics review at Karolinska Institutet, Sweden [2008/919-31]. Detailed information about the study was provided verbally to the participants. The interviews were conducted after obtaining verbal informed consent. The respondents' identities were kept confidential. If a woman scored more than16 on the EPDS during the study, we referred her to the psychiatric department of a nearby hospital.

## Results

### Sample profile

The final sample included 720 women in the third trimester of pregnancy with a mean age of 25 years. Almost 60% of the study sample was literate. The average level of schooling was 3.8 years. More than 90% of the women were homemakers. One third of the women were involved in income-earning activities, such as weaving, handicraft, or poultry rearing. Almost 60% of the respondents owned less than 50 decimals of land (100 decimals = 1 acre), indicating poor economic status in terms of ownership of land. The median daily per capita household expenditure on food was BDT 31.25 (USD 0.45) (Table 1). Approximately 97% of the women were Muslim (data not shown).

**Table 1:** Socioeconomic, obstetric, family support, and partner violence profile of the women at third trimester of pregnancy (in percent unless otherwise specified)

Baseline characteristics	%
**Socioeconomic profile**	
Age	
- ≤19 years	21.8
- 20-34 years	69.9
- ≥35 years	8.3
- Mean age (±SD)	24.58 (±6.15)
Literacy (can read and write)	58.9
Years of schooling	
- No schooling	38.8
- 1-5	32.8
- 6-10	22.8
- >10	5.7
- Mean years of schooling (±SD)	3.8 (±3.9)
Husbands' literacy	45.3
Involved in income-earning activities	29.7
Total land owned by household (in decimals^3^)	
- No land	2.8
- 1-49	59.7
- 50-99	13.2
- ≥100	24.3
Median per capita daily household expenditure on food (BDT)^1 ^(range)	31.25 (8.95-105.36)

**Obstetric information**	
Mean number of children (±SD)	1.9 (±1.9)
Child's death	23.3
Planned current pregnancy	51.1
Received antenatal care	44.0

**Family support **	
Poor relationship with husband	23.9
Poor relationship with mother-in-law^2 ^	48.1
Practical support received during pregnancy	80.7

**Intimate partner violence **	
Experienced multiple acts of physical violence	33.8
Experienced forced sex	79.2
Experienced physical violence during pregnancy	18.1

**Total**	720

^1^BDT 68 = USD 1 during the study period

^2^N = 619 as 101 mothers-in-law were dead before the interview date

^3^100 decimals = 1 acre

### Obstetric profile

Almost 28% of respondents were primiparas. The mean number of children was 1.9. Almost one fourth of mothers had experienced the death of 1-3 children including miscarriage and stillbirth.

### Family support and intimate partner violence

Seven in ten participants reported being the victim of at least a single act of physical intimate partner violence (data not shown), one third of multiple acts, and eight in ten experienced being forced into having sex with their spouse. Eighteen percent reported physical violence during the current pregnancy. Despite the high prevalence of reported intimate partner violence, only twenty-four percent rated their relationship with their husband as bad. Half of the women reported that they had a bad relationship with their mother-in-law. However, almost eighty percent of the participants stated that they had experienced practical support during the current pregnancy from family members (Table 1). The source of the support in most cases was the husband or the mother-in-law. One in ten women reported a previous history of depressive symptoms (data not shown).

### Prevalence of antepartum depressive and anxiety symptoms

Of the 720 participants assessed at the third trimester of pregnancy, 132 had EPDS scores ≥10, indicating an ADS point prevalence of 18.3% (CI_95% _15.9%-20.7%), mean score 12.3 (SD 2.8). Similarly, the point prevalence of AAS (STAI ≥45) was 29.4% (CI_95% _26.1%-32.7%), mean score 49.6 (SD 4.6). Twenty-three women (3.4%) had both ADS and AAS (data not shown).

### Determinants of ADS and AAS

A number of independent factors potentially associated with ADS and AAS were examined. These were organized into factors defined as socioeconomic, family support, intimate partner violence, obstetric, and previous history of depressive symptoms (Tables 2 and 3).

**Table 2:** Logistic regression model showing associated factors of antepartum depressive symptoms among women in a rural area of Bangladesh

Associated factors	Depressed N = 132 (%)	Non-depressed N = 588 (%)	Adjusted OR	CI_95%_	Wald
**Socioeconomic indicators **					
Age					
<20 years	22 (16.7)	135 (23.0)	1		
20-34 years	90 (68.2)	413 (70.2)	1.48	0.71-3.06	1.09
≥35 years	20 (15.2)	40 (6.8)	3.00	1.12-8.01	4.79
Literacy					
Cannot read and write	75 (56.8)	221 (37.6)	1		
Can read and write	57 (43.2)	367 (62.4)	0.59	0.37-0.95	4.68
Poor household economic status					
≥BDT 31.25	59(44.7)	301 (51.2)	1		
<BDT 31.25	73 (55.3)	287 (48.8)	0.91	0.57-1.45	0.16
Involve in income-generating activities					
No	82 (62.1)	424 (72.1)	1		
Yes	50 (37.9)	164 (27.9)	1.24	0.78-1.97	0.80

**Family Support**					
Relationship with husband					
Good	74 (56.5)	473 (80.4)	1		
Poor	57 (43.5)	115 (19.6)	2.23	3.37-3.62	10.49
Practical support during pregnancy					
No support received	38 (28.8)	101 (17.2)	1		
Support received	94 (71.2)	487 (82.8)	0.61	0.36-1.01	3.73

**Intimate partner violence**					
Experienced forced sex ever					
No	14 (10.6)	136 (23.1)	1		
Yes	118 (89.4)	452 (76.9)	1.95	1.01-3.75	3.95
Experienced multiple acts of physical violence					
No	61 (46.2)	416 (70.7)	1		
Yes	71 (53.8)	172 (29.3)	1.69	1.02-2.80	4.08

**Obstetric indicators**					
Parity					
Multipara	107 (81.1)	413 (70.2)	1		
Primipara	25 (18.9)	175 (29.8)	1.33	0.66-2.70	0.62
History of child death					
No	93 (70.5)	459 (78.1)	1		
Yes	39 (29.5)	129 (21.9)	1.15	0.69-1.92	0.29
Current pregnancy planned					
No	81 (61.4)	271 (46.1)	1		
Yes	51 (38.6)	317 (53.9)	0.76	0.48-1.21	1.32

**Previous history of mental illness^1 ^**					
No	86 (69.9)	505 (92.2)	1		
Yes	37 (30.1)	43 (7.8)	4.62	2.72-7.85	31.91

^1^N = 671, because this information was collected during 2-3 months postpartum when 49 women were lost to follow-up due to out-migration, multiple birth, still birth, neonatal death, maternal and child death.

**Table 3:** Linear regression models examining associated factors of general anxiety symptoms among rural Bangladeshi women during third trimester of pregnancy (N = 720)

Associated factors	B	Std. error	p value
**Socioeconomic indicators**			
Age*	-0.035	0.051	0.499
Literacy (yes = 1, no = 0)	-2.079	0.543	0.000
Involved in income-earning activity (yes = 1, no = 0)	0.332	0.543	0.534
Per capita daily household	-0.077	0.019	0.000
expenditure on food*			

**Family support**			
Relationship with husband (poor = 1, good = 0)	2.344	0.632	0.000
Practical support during pregnancy (yes = 1, no = 0)	-1.447	0.626	0.021

**Intimate partner violence**			
Number of acts of physical violence experienced*	0.521	0.202	0.010
Ever experienced forced sex (yes = 1, no = 0)	0.769	0.582	0.187
Experienced violence during pregnancy (yes = 1, no = 0)	1.612	0.714	0.024

**Obstetric indicator**			
Parity (primipara = 1, multipara = 0)	-0.378	0.699	0.589
History of child death (yes = 1, no = 0)	0.639	0.619	0.302
Current pregnancy planned (yes = 1, no = 0)	-0.753	0.518	0.146
Poor household economy × poor relationship with husband	0.083	0.018	0.000

Total R^2^	R^2 ^= 0.19 R^2 ^(adjusted) = 0.18		

*Continuous scale

After adjusting the multivariate logistic regression model, ADS was significantly associated with six factors (Table 2). These were older age (≥35 years) (OR = 3.0; CI_95% _= 1.12-8.01), literacy (OR = 0.59; Cl_95% _= 0.37-0.95), poor relationship with husband (OR = 2.23; Cl_95% _= 3.37-3.62), forced sex ever (OR = 1.95; Cl_95% _= 1.01-3.75), physical violence (OR = 1.69; Cl_95% _= 1.02-2.80), and previous history of depressive symptoms (OR = 4.62; Cl_95% _= 2.72-7.85). Using the Wald estimator, a previous history of depressive symptoms showed the strongest association with ADS, followed by poor relationship with husband, older maternal age, literacy, physical violence, and forced sex. No significant interaction between the explanatory variables was found. The models indicated almost the same log-likelihood ratio (-540.23) after excluding outliers (-538.98). A Hosmer-Lemeshow test (p = 0.794) indicated that the model fit the data well.

The adjusted linear regression model for anxiety showed similar results (Table 3). The model shows that general anxiety (STAI-trait) was inversely associated with literacy, per capita daily household expenditure on food, and practical support during pregnancy; it was directly associated with poor relationship with husband, physical violence ever, and violence during pregnancy. The interaction between poor household economy and poor relationship with husband was significant. All the considered associated factors explained 19% of the variance. No violation of assumption was observed.

## Discussion

The main finding of this study suggests that ADS is prevalent in almost one in five women, and AAS in one third of the women, in the last trimester of pregnancy in a rural area of Bangladesh. The women's literacy, their poor household economy (anxiety only), poor relationships with husbands, and partner violence showed strong associations with ADS and AAS. A previous history of depressive symptoms was found to have the strongest association with ADS.

The prevalence of ADS (18%) and AAS (29%) are in agreement with previous reports from both high-income countries (ADS 12% [8], AAS 24% [9]) and low-income countries, with 20% in Pakistan (both ADS and AAS) [11], and 10%-20% in Brazil [25,26]. A previous study in Bangladesh had reported a higher prevalence of ADS, 33%, which may be due to a recruitment of women at a later stage of pregnancy, in this case 34-35 weeks, compared to the current study [16]. The prevalence of depression may be affected by the time point in pregnancy at which symptoms are assessed [8], types of instrument used i.e. use of standard clinical interviews or screening tools [27], and different cut-off points on screening tools [28]. For instance, the higher prevalence of antepartum anxiety (54%) and depressive (37%) symptoms reported in Hong Kong compared to our study may be due to that the sample was selected from an antenatal clinic who went through screening interviews using the Hospital Anxiety and Depression Scale which was developed especially for medical patients [29].

The direct association between poverty and depression is well documented in high-income countries [30,31], and the evidence based on this association from low-income countries is growing [32]. A recent study in Pakistan found a positive association between lower household wealth and antepartum depression/anxiety [11]. The relationship between poverty (indicated by low level of education, minimum wage, low household income, and husband's unemployment) and mental disorder have been elucidated in a recent review of studies from six low- and middle-income countries [33]. This association was also observed in our data in cases of AAS, but not for ADS. Bangladesh is a socially disadvantaged country where 40% of the population lives below poverty line [34]. Poverty may be a general source of anxiety for pregnant women in terms of the family's growing financial needs with the increased number of children and the need to ensure food and education for the children. However, because of the homogeneity of poverty, this does not necessarily lead to depressive symptoms among women expecting a new member of the family.

The positive effect of literacy was pronounced in our study on the outcomes of both ADS and AAS and is consistent with the findings from Brazil, Chile, Indonesia, and Pakistan [33]. Literate women may have good social networks and social support, which has been identified as a protective factor in previous research [35]. Francis et al [36] and Weiss et al [37] explain that literacy gives individuals a sense of improved self-esteem or self-efficacy, enhances their feelings of self-worth, diminishes feelings of shame, and in turn, reduces depressive and anxiety symptoms. In contrast, a U.S.-based study highlighted education as a risk factor, explaining that highly educated individuals are more sensitive to and/or are not embarrassed about admitting depressive symptoms [38]. Consistently with previous research, a history of previous depression was a significant contributory factor for the development of perinatal depression [35,39].

Bangladesh has one of the highest rates of violence in the world [40]. Of the women in the current study, 34% reported being abused physically by multiple (three or four) acts and 80% sexually (forced into having sex) by their husbands, results that compare well with the level of violence (two fifths) among rural women found in another study in Bangladesh [41]. Yet only about a quarter of the women reported poor relationships with their husband in general. It is reported in previous research that rural women in Bangladesh do not necessarily recognize certain acts (such as a slap or shove) as violence and that such violent behavior is considered to be the husband's prerogative [42]. Hence, the single acts of violence reported by 70% of the women in this study may be viewed by them as corrective punishment by their husband [43]. A WHO multicountry study indicated that 50%-90% of the women accepted violence by their husbands under certain circumstances as normal, such circumstances being if a woman goes out without informing her husband, neglects their children, argues with him, refuses to have sex with him, or burns the food [19]. Gender-based violence has been described as the single most important predictor of depression [44] and anxiety [11] in women. This evidence is well documented in high-income countries [31] and is growing in low-income countries [11,16,25]. Violence during pregnancy results in adverse consequences for fetal and maternal survival [25]. The current study identifies intimate partner violence, particularly physical violence, as contributing to ADS and AAS, and violence during pregnancy as contributing to AAS. Our findings also strengthen the results of the only study in Bangladesh identifying partner violence and partner relationship as predictors of ADS [16]. Despite the relatively low percentage of women reporting poor relationships with their husbands particularly in terms of the prevalence of intimate partner violence, this study shows that poor marital relationship does have a significant impact on reporting both ADS and AAS. An even more pronounced effect was observed in the case of AAS, when poor partner relationship interacted with poor household economy.

This study identified practical support from the mother-in-law and husband as protective against anxiety and depressive symptoms among pregnant women. These findings reflect the observations of other researchers that family/social support during pregnancy plays a significant role in predicting women's emotional status in the antepartum period [45]. Support from family members may be of special significance in the context of traditional South Asian societies. In these societies, the newlywed couple usually becomes part of the husband's extended family, including his parents and siblings. Rarely do couples set up homes of their own. In such cases, the newlywed woman's mother-in-law is the matriarch who holds effective power and control over household matters. Under her guidance and supervision, the daughter-in-law is expected to carry out all instructions and household chores [46]. In this context, receiving support from the mother-in-law is a sign of approval and a source of confidence and, hence, protective against antenatal depressive and anxiety symptoms.

The current study has utilized a widely used and locally validated scale to measure postpartum depressive symptoms [22] that is also routinely used in the antepartum period and in clinical and community settings [21]. However, the anxiety scale used in the study is not validated for low-income countries such as Bangladesh; thus, the STAI was used as a continuous scale in a linear regression. The cutoff at a ≥45 percentile in the study to report prevalence is similar to that used in high-income countries [24]. A further limitation of the study is that it was conducted in two subdistricts of rural Bangladesh and does not represent the urban scenario. Although the findings cannot be generalized even for other rural areas of the country, the community-based sample is likely to be indicative of the situation among rural women. As the study was cross-sectional in nature, issues related to causality cannot be addressed. Additionally several important variables were not controlled for, such as physical illness, gynecological morbidities, previous infertility, complications during pregnancy (hypertension, preeclampsia, bleeding), and smoking. It is very difficult to set a cutoff for any psychological state because of its subjective nature which may over- or under-report the prevalence of ADS and AAS. In the case of reporting physical violence, women may not have reported the actual scenario due to its sensitive nature.

## Conclusions

Our study confirms that depression and anxiety are common during pregnancy, and that illiteracy, intimate partner violence, bad relationships with husbands, a lack of practical support, and previous depressive symptoms are independent factors associated with ADS and AAS. The study indicates the necessity of integrating mental health with existing maternal and child health care in Bangladesh. All those concerned with antenatal care need to pay attention and screen for depressive and anxiety symptoms. Health care professionals, including community health workers, need to enquire about the relevant risk factors as part of their overall assessment. Policies aimed at referring women with depressive symptoms to the nearest medical college hospital where psychological treatment is freely provided may help mothers in receiving appropriate support. Providing practical support to women during pregnancy, particularly those with a previous history of depression, reducing gender-based violence, and supporting women in poor partner relationship are important preventive strategies to adopt at the community level. Local development organizations, women's groups, and government health care settings have important roles to play in improving the mental health of pregnant women.

## Abbreviations

ADS: Antepartum depressive symptoms; AAS: Antepartum anxiety symptoms; WHO: World Health Organization; EPDS: Edinburgh Postnatal Depression Scale; STAI: State Trait Anxiety Inventory.

## Competing interests

The authors declare that they have no competing interests.

## Authors' contributions

All authors (HEN, ZNK, YF, ME) participated in the planning and conception of the research questions and the study design. ME was the principal investigator of the study and primarily conceptualized the research. HEN was responsible for data collection, and HEN and ME were responsible for analyzing the data. HEN drafted the article, and all authors participated in interpreting the data and revising the manuscript. All authors read and approved the final manuscript.

## Pre-publication history

The pre-publication history for this paper can be accessed here:

http://www.biomedcentral.com/1472-6874/11/22/prepub

## Supplementary Material

Additional file 1 **English translation of the original Bangla questionnaire**. Click here for file
