# Supplementary material for: Prevalence and associated factors of depressive and anxiety symptoms during pregnancy: A population based study in rural Bangladesh
Source: BMC Womens Health. 2011 Jun 2;11:22. doi: 10.1186/1472-6874-11-22 (PMC3117808; doi:10.1186/1472-6874-11-22)
Supplement: Additional file 1 — English translation of the original Bangla questionnaire. [file 1472-6874-11-22-S1.DOC]

**BRAC-Karolinska Institute Joint Research Project**

**Impact of maternal perinatal depressive symptoms on infant growth and health in Bangladesh**

**Survey 2008**

Day Month Year

**ID no.: Date:**

Respondent’s Name: --------------------- Husbands’s Name: -----------------------------------------

House Name: ----------------------------- Household no.:

Para: --------------------------- Union: -------------------------

Village: ---------------------------- Thana / Upazilla :-------------------

District-----------------------------

**Respondent’s status:**

1

Pregnant woman (7-9 months 7 days/ 40 weeks in pregnancy)

2

Postpartum mother (Mother of child 2-3 months old)

3

Postpartum mother (mother of child 6 months old)

Day Month Year

Day Month Year

First date of last Expected delivery date:

menstruation:

## INFORMED CONSENT

I work for an organization named BRAC. At present BRAC is collecting information to understand the impact of maternal depression during pregnancy and the postpartum period on child growth and health. As you know this topic has been fairly neglected in Bangladesh. The present research would work on maternal depression and would help in developing ways of treatment seeking in the future. I therefore, would like to ask you some questions on this topic. I assure you that everything you say will be kept confidential. If you want, you can refuse to answer the questions. You will not face any problems for that. If you agree, I will start asking questions.

Do you have any further queries regarding our survey? May I start the interview?

1

2

Respondent agreed Respondent did not agree

If you have any queries in the future please contact the numbers given below.

Dr. Hashima-e-Nasreen – Mobile number

Ms. Sarawat Rashid – Mobile number

**Name of interviewer: ---------------------------------------- Date:**

**SCRUTINIZED BY --------------------------------------- SPOT CHECKED BY --------------------------**

**CROSS CHECKED BY --------------------------------- RE-INTERVIEWED BY ----------------------**

**EDITED BY ------------------------------------------ CODED BY --------------------------------------**

**Section A: Socio Economic Information**

**1. Socio**-economic

| Status of respondent | Age  (years) | Household head (Who takes decision for the HH)  M= 1  F= 2 | No. of members of the HH | Religion | Marital Status | Can read or write  1= Yes;  2= No | Educational qualification (passed which class) | Income earner  1= yes;  2= No | Main Occupation  (According to respondent) |
| --- | --- | --- | --- | --- | --- | --- | --- | --- | --- |
| (1) | (2) | (3) | (4) | (5) | (6) | (7) | (8) | (9) | (10) |
| Women |  |  |  |  |  |  |  |  |  |
| Husband |  |  |  |  | ___ |  |  |  |  |

| **(5) Religion**  1= Islam; 2 = Hindu;  77 **= Others .............................** (Please specify)  **(6) Marital status**  1 **=** Married, 2 = Unmarried; 3 = Divorced;  4 = Separated; 5 = Widow  **(8) Education**  Write down the class passed.  No class passed/no schooling=00  10= SSC, 12= HSC, 14= graduate, 16=masters / Doctor/ Engineer/ lawer *,* 55 = hafeji/kawmi/khariji 99 = Don’t know | **(10) Main occupation**  1 = Agriculture  2 = fishermen  3 = Household based work (Weaving/handicrafts)  4 = Cattle, poultry rearing  5 = Construction worker  6 = Skilled Labor (Carpenter, Blacksmith, Goldsmith, Potter)  7 = driver (Truck, bus)  8 = Rickshaw/ Van driver | 9 = Day laborer  10 = Service  11 = Teacher  12 = Business  13 =Small business (milkman, fruits or vegetable seller, retailer )  14 = Service (Doctor, Engineer, lawer)  15 = Unemployed  16 = Housewife  77= Others.......................................  (Please specify) |
| --- | --- | --- |

**2. Anthropometric measurement of mothers:**

**Please note the correct measurement**

| **Height**  **cm** | **Weight**  **kg** | **MUAC**  **mm** |
| --- | --- | --- |
|  |  |  |

**3. NGO involvement and land holding**

| BRAC Member  1= Yes  2= No | If yes, then its type | NGO member other then BRAC  1= Yes; 2= No | If yes, name of NGO  * | Land including homestead (Decimal) | Does anybody in the household sells manual labor  1= Yes; 2= No | BRAC Eligible  1= Yes; 2= No |
| --- | --- | --- | --- | --- | --- | --- |
| (1) | (2) | (3) | (4) | (5) | (6) | (7) |
|  |  |  |  |  |  |  |

| **(2) BRAC Samiti**  1=TUP; 2=IGVGD; 3=Dabi; 4=Unnoti; 5=Progoti; 99=Don’t know | | **(5) Quantity of Land**  (Write in decimal : For no land write 00 ) | **(7)** land less than 50 decimals and at least 1 member of the household sells manual labor for 100 days. |
| --- | --- | --- |

*****Multiple answers considered

**4. Latrine in the household**

| Season | Type | Ownership |
| --- | --- | --- |
| Rainy (1) |  |  |
| Dry (2) |  |  |

| **Type:** 1= Hither and thither, 2= Open pit, 3= Pit, 4= Ring slab (water sealed) 5= Ring slab (not water sealed), 6= Sanitary (Water seal and septic tank), 7= River/ canal/ pond, 8= Road side, 9= Bush, 10= Hanging,  77= Others.......................................... ....(specify) | **Ownership:** 1= Own, 2= Joint (with ownership), 3= Joint (without ownership), 88= not applicable (river/ canal/ pond/ bush/ roadside/ hither and thither) |
| --- | --- |

**5**. Access to safe water

| Use | SourceDry season | SourceRainy season |
| --- | --- | --- |
| Drinking |  |  |
| Cooking |  |  |
| Washing utensils |  |  |
| Washing clothes |  |  |
| Bathing |  |  |
| Water used after defecation |  |  |

| 1= Own tube-well, 2= Shared tubewell/ Others tube-well, 3= Supply water/ Tap water/Pipe water, 4= Public tube-well, 5= River/ canal/pond/ Ditch, 6= well, 77= Others ................................... (Specify) |
| --- |

**6**. Food Consumption per week (By the household)

| **List of food items consumed in the last 7 days** | **Yes = 1, No =2** | **Quantity/ week** | **Cost** |
| --- | --- | --- | --- |
| Meat |  |  |  |
| Fish |  |  |  |
| Egg |  |  |  |
| Milk |  |  |  |
| Green vegetables |  |  |  |
| Other vegetables |  |  |  |
| Oil |  |  |  |
| Sugar/ Sweets |  |  |  |
| Fruits |  |  |  |
| Pulses |  |  |  |
| Rice/ *Chapati* (bread)/ flour/ *suji* |  |  |  |
| Others..............................77 |  |  |  |

**(Interviewer to carry a list of the current market prices of the above items)**

##### 6.2 Total expenditure on food per week............................................................................. (Taka)

##### Section B: Social support network

**7. Questions related to social support network**

| **Sl .NO** | **Question** | Answer | **Code** | **Skip to** |
| --- | --- | --- | --- | --- |
| **7.1** | How is your relationship with your husband?  **(Read out the answers repeatedly)** | Good  In-between  Bad  Not applicable (If divorced, abandoned, separated or dead) | 1  2  3  88 |  |
| **7.2** | How is your relationship with your mother-in-law?  **(Read out the answers repeatedly)** | Good  In-between  Bad  Not applicable (e.g. if dead) | 1  2  3  88 |  |
| **7.3** | During your current pregnancy, do you receive practical help in daily activities from others? | Yes  No | 1  2 | **8.17** |
| ***7.4** | Who helps you in your daily activities? | Husband  Father in law  Mother inlaw  Relatives (In-laws)  Father  Mother  Relatives (paternal-maternal)  Neighbour  Friend  Others..............................(specify) | 1  2  3  4  5  6  7  8  9  77 |  |
| **7.5** | Do you think that this help is enough? | Yes  No | 1  2 |  |
| **7.6** | During your current pregnancy, do you receive emotional support from others? | Yes  No | 1  2 | **8.20** |

| **7.7** | Who supports you emotionally? | Husband  Father in law  Mother inlaw  Relatives (In-laws)  Father  Mother  Relatives (paternal-maternal)  Neighbour  Friend  Community leader  Religious leader *(Pir,fakir,hakim,*  *moulana,purohit,thakur)*  Sastha Kormi  Sastha Shebika  Trained traditional birth attendant (TTBA)  Trained birth attendant (TBA)  Family welfare visitor (FWV)  Village doctor  *Kabiraj* (Traditional healer)  Homeopath doctor  Others..............................(specify) | 1  2  3  4  5  6  7  8  9  10  11  12  13  14  15  16  17  18  19  77 |  |
| --- | --- | --- | --- | --- |
| **7.8** | Do you think that this emotional support is enough? | Yes  No | 1  2 |  |

##### Section C: Intimate partner violence

##### 8. Questions related to intimate partner violence

| **Sl .NO** | **Question** | **Answer** | **Code** | **Skip to** |
| --- | --- | --- | --- | --- |
| 8.1 | Has your husband ever slapped you or thrown anything at you to hurt you? | Yes  No | 1  2 |  |
| 8.2 | Has your husband ever shoved you to the ground? | Yes  No | 1  2 |  |
| 8.3 | Has your husband ever punched you? | Yes  No | 1  2 |  |
| 8.4 | Has your husband ever kicked you or dragged you on the ground? | Yes  No | 1  2 |  |
| 8.5 | Has your husband ever forced you to have sexual intercourse? | Yes  No | 1  2 |  |
| 8.6 | Do you think it is a sin to refuse your husband sexual intercourse when you don’t want to? | Yes  No | 1  2 |  |
| 8.7 | Has your husband slapped, shoved, punched or kicked you during your current pregnancy? | Yes  No | 1  2 |  |

# Section D: Reproductive Health Information

9. Questions related to reproductive health information

| **Sl.no.** | **Question** | **Answer** | **Code** | **Skip to** |
| --- | --- | --- | --- | --- |
| **9.1** | Number of children given birth to and their sex. | ............. Number (Alive+Dead)  M__________ F_______  None (Alive/Dead) | **55** | **10.4** |
| **9.2** | Age of live children (years) | First------------------- years  Don’t know  Second ----------------- years  Don’t know  Third ------------------ years  Don’t know  Fourth---------------- years  Don’t know  Fifth----------------- years  Don’t know  ............................... | **99**  **99**  **99**  **99**  **99** |  |
| **9.3** | How many of your children have died and at what ages? | Total ........................ number  Intra-uterine death ...........number  During delivery . .. ........ .number Neonate( 28 days) . . . . .number Within 1 year . ... . number  Within 2 years . . . . . . .number  Within 3-5 years . ...........number  Others . . . . . . . . . . . . . . .  (Please specify) | **77** |  |
| **9.4** | Did you plan your current pregnancy? | Yes  No | **1**  **2** |  |
| **9.5** | Are you happy with this pregnancy? | Yes  No | **1**  **2** |  |
| **9.6** | Do youreceive antenatal care (ANC)? | Yes  No | **1**  **2** | **10.21** |
| **9.7** | During this pregnancy how many times have you received antenatal check-up (ANC)? | . . . . . . . . . . Times  Don’t remember | **44** |  |
| ***9.8** | What services did you receive? | Pulse examination  Blood pressure check  Weight measurement  Height measurement  Anemia test  Blood test  Urine test  Foetal position  Foetal heart beat  Ultrasonogram  Others......................................  (Specify)  Don’t remember  Don’t know | **1**  **2**  **3**  **4**  **5**  **6**  **7**  **8**  **9**  **10**  **77**  **44**  **99** |  |
| ***9.9** | Where/ to whom did you go for this checkup? | District Hospital  Upazilla/Thana health Complex  Maternal and child welfare centre (MCWC)  BRAC *Shusasthya*  Private clinic  MBBS Doctor (GP)  Shasthya Kormi (SK)  Trained traditional birth attendant (TTBA)  Trained birth attendant (TBA)  Family welfare visitor (FWV)  Village doctor  *Kabiraj* (traditional healer)  Homeopathic  Others......................................  (Specify)  Don’t remember  Don’t know | **1**  **2**  **3**  **4**  **5**  **6**  **7**  **8**  **9**  **10**  **11**  **12**  **13**  **77**  **44**  **99** |  |

***Multiple answers considered**

**Section E: Maternal depression**

**Directon:** Please ask a pregnant woman. Correctly number the answers, which depict how the woman feels not only today but also how she felt in the past seven days. There is no right or wrong answer. (Read the questions and answers repeatedly till the respondent clearly understands them. Circle the score depending on the answers provided.)

**10. Scale for measuring depression using Edinburgh Postnatal Depression Scale (EPDS)**

| **SI.NO** | Question | **Answer** | **Score** |
| --- | --- | --- | --- |
| **10.1** | In the past 7 days have you been able to laugh and see the funny side of things? | As much as I always could | **0** |
|  |  | Not quite so much now | **1** |
|  |  | Definitely not so much now | **2** |
|  |  | Not at all | **3** |
| **10.2** | In the past 7 days have you looked forward with enjoyment to things? | As much as I ever did | **0** |
|  |  | Rather less than I used to | **1** |
|  |  | Definitely less than I used to | **2** |
|  |  | Hardly at all | **3** |
| **10.3** | In the past 7 days have you blamed yourself unnecessarily when things went wrong? | Yes, most of the time | **3** |
|  |  | Yes, some of the time | **2** |
|  |  | Not very often | **1** |
|  |  | No, never | **0** |
| **10.4** | In the past 7 days have you been anxious or worried for no good reason? | No, not at all | **0** |
|  |  | Hardly ever | **1** |
|  |  | Yes, sometimes | **2** |
|  |  | Yes, very often | **3** |
| **10.5** | In the past 7 days have you felt scared or panicky for no good reason? | Yes, quite a lot | **3** |
|  |  | Yes, sometimes | **2** |
|  |  | No, not much | **1** |
|  |  | No, not at all | **0** |
| **10.6** | In the past 7 days have you felt that things have been getting on top of you? | Yes, most of the time I haven’t been able to cope at all | **3** |
|  |  | Yes, sometimes I haven't been coping as well as usual | **2** |
|  |  | No, most of the time I have coped quite well | **1** |
|  |  | No, I have been coping as well as ever | **0** |
| **10.7** | In the past 7 days have you felt so unhappy that you had difficulty sleeping? | Yes, most of the time | **3** |
|  |  | Yes, sometimes | **2** |
|  |  | Not very often | **1** |
|  |  | No, not at all | **0** |
| **10.8** | In the past 7 days have you felt sad or miserable? | Yes, most of the time | **3** |
|  |  | Yes, quite often | **2** |
|  |  | Not very often | **1** |
|  |  | No, not at all | **0** |
| **10.9** | In the past 7 days were you so unhappy that you have been crying? | Yes, most of the time | **3** |
|  |  | Yes, quite often | **2** |
|  |  | Only occasionally | **1** |
|  |  | No, never | **0** |
| **10.10** | In the past 7 days, have thoughts of harming yourself occured? | Yes, quite often | **3** |
|  |  | Sometimes | **2** |
|  |  | Hardly ever | **1** |
|  |  | Never | **0** |
| Score Range: 0-30 Total = | | | |

The following questions are related to respondent’s health seeking behavior. Ask questions 10.11 to 10.16 to respondents who score 10 or more on the EPDS. **If the score on the EPDS is 16 or more ask for women’s permission to inform the local health worker. If the respondent scores 3 on questions 10 in EPDS, ask for permission to inform the local health worker and also ask them the SCAN questionnaire.** Otherwise go to 11.Section F.

| **Sl.No.** | **Question** | **Answer** | **Code** | **Skip to** |
| --- | --- | --- | --- | --- |
| **10.11** | Have you sought any help for depression? | Yes  No | **1**  **2** | **12.1** |
| **10.12** | Where did you seek help? | MBBS Doctor (GP)  Homeopath  Pharmacist  Village doctor  *Kabiraj* (traditional healer)  Religious treatment (*Ohjha*;*Pir,fakir, hakim,moulana,purohit,thakur)*  Medical college hospital  District hospital  Upazilla/Thana health Complex  Family welfare centre/ Maternal and child welfare centre (MCWC)  Local *Shasthaya Karmi*  Don’t know  Others......................................  (Specify | **1**  **2**  **3**  **4**  **5**  **6**  **7**  **8**  **9**  **10**  **11**  **12**  **99**  **77** |  |
| **10.13** | What kind of help did you receive? | Did not receive any help  Counseling  Medicine  Exorcise evil spirits (*jhar phuk*)  Chanted water/chanted oil  Amulet  Herbs  Others......................................  (Specify) | **0**  **1**  **2**  **3**  **4**  **5**  **6**  **77** |  |
| **10.14** | Have you ever faced depressive symptoms before? | Yes  No | **1**  **2** | **12.1** |
| **10.15** | If yes, where did you seek help? | MBBS Doctor (GP)  Homeopath  Pharmacist  Village doctor  Kabiraj (traditional healer  Religious treatment (*Ohjha*; *Pir, fakir, hakim, moulana, purohit, thakur)*  Medical college hospital  District hospital  Upazilla/Thana health Complex  Family welfare centre/ Maternal and child welfare centre (MCWC)  Local *Shasthaya Karmi*  Don’t know  Others......................................  (Specify | **1**  **2**  **3**  **4**  **5**  **6**  **7**  **8**  **9**  **10**  **11**  **12**  **99**  **77** |  |

| **10.16** | What kind of help did you take? | Did not receive any help  Counseling  Medicine  Exorcise evil spirits (*Jhar-phuk*)  Chanted water/chanted oil  Amulet  Herbs  Others......................................  (Specify) | **0**  **1**  **2**  **3**  **4**  **5**  **6**  **77** |  |
| --- | --- | --- | --- | --- |

**Section F: Maternal general anxiety**

**11. Scale for measuring maternal general anxiety using State and Trait Anxiety Inventory**

**Direction:** Please read the following statements and correctly number how the respondent generally feels. There is no right or wrong answer. (Read the questions and answers repeatedly till the respondent clearly understands them. Circle the score depending on the answers provided.)

| **SI. NO** | **Question** | **Answer** | **Score** |
| --- | --- | --- | --- |
| **11.1** | You generally feel pleasant | Almost never | **1** |
|  |  | Sometimes | **2** |
|  |  | Often | **3** |
|  |  | Almost always | **4** |
| **11.2** | You generally feel nervous and restless | Almost never | **1** |
|  |  | Sometimes | **2** |
|  |  | Often | **3** |
|  |  | Almost always | **4** |
| **11.3** | You generally feel satisfied with yourself | Almost never | **1** |
|  |  | Sometimes | **2** |
|  |  | Often | **3** |
|  |  | Almost always | **4** |
| **11.4** | You generally wish you could be as happy as others seem to be | Almost never | **1** |
|  |  | Sometimes | **2** |
|  |  | Often | **3** |
|  |  | Almost always | **4** |
| **11.5** | You generally feel like a failure | Almost never | **1** |
|  |  | Sometimes | **2** |
|  |  | Often | **3** |
|  |  | Almost always | **4** |
| **11.6** | You generally feel rested | Almost never | **1** |
|  |  | Sometimes | **2** |
|  |  | Often | **3** |
|  |  | Almost always | **4** |
| **11.7** | You are generally ‘calm, cool and collected’ | Almost never | **1** |
|  |  | Sometimes | **2** |
|  |  | Often | **3** |
|  |  | Almost always | **4** |
| **11.8** | You generally feel that difficulties are piling up so that you cannot overcome them | Almost never | **1** |
|  |  | Sometimes | **2** |
|  |  | Often | **3** |
|  |  | Almost always | **4** |
| **11.9** | You generally worry too much over something that really doesn’t matter | Almost never | **1** |
|  |  | Sometimes | **2** |
|  |  | Often | **3** |
|  |  | Almost always | **4** |
| **11.10** | You are happy | Almost never | **1** |
|  |  | Sometimes | **2** |
|  |  | Often | **3** |
|  |  | Almost always | **4** |
| **11.11** | You generally have disturbing thoughts | Almost never | **1** |
|  |  | Sometimes | **2** |
|  |  | Often | **3** |
|  |  | Almost always | **4** |
| **11.12** | You generally lack self confidence | Almost never | **1** |
|  |  | Sometimes | **2** |
|  |  | Often | **3** |
|  |  | Almost always | **4** |
| **11.13** | You generally feel secure | Almost never | **1** |
|  |  | Sometimes | **2** |
|  |  | Often | **3** |
|  |  | Almost always | **4** |
| **11.14** | You generally make decisions easily | Almost never | **1** |
|  |  | Sometimes | **2** |
|  |  | Often | **3** |
|  |  | Almost always | **4** |
| **11.15** | You generally feel inadequate | Almost never | **1** |
|  |  | Sometimes | **2** |
|  |  | Often | **3** |
|  |  | Almost always | **4** |
| **11.16** | You are generally content | Almost never | **1** |
|  |  | Sometimes | **2** |
|  |  | Often | **3** |
|  |  | Almost always | **4** |
| **11.17** | Some unimportant thoughts generally run through your mind and bother you | Almost never | **1** |
|  |  | Sometimes | **2** |
|  |  | Often | **3** |
|  |  | Almost always | **4** |
| **11.18** | You generally take disappointments so keenly that you can’t put them out of your mind | Almost never | **1** |
|  |  | Sometimes | **2** |
|  |  | Often | **3** |
|  |  | Almost always | **4** |
| **11.19** | You are a steady person | Almost never | **1** |
|  |  | Sometimes | **2** |
|  |  | Often | **3** |
|  |  | Almost always | **4** |
| **11.20** | You get into a state of tension or turmoil as you think over your recent concerns and interests | Almost never | **1** |
|  |  | Sometimes | **2** |
|  |  | Often | **3** |
|  |  | Almost always | **4** |
| Total Score Range: 20-80: | | | |

Scale for assessing depressive disorder associated with psychotic symptoms using Schedule for Clinical Assessment in Neuropsychiatry (SCAN)

**Direction: Please ask those respondents who had scored 3 in question 10 of EPDS.**

| **SI.NO** | **Question** | Answer | Score |
| --- | --- | --- | --- |
| **1** | Do you feel a change in the appearance of things? | No | **0** |
|  |  | Yes | **1** |
| **2** | Have you had the feeling that something odd is going on that you can’t explain? | No | **0** |
|  |  | Yes | **1** |
| **3** | Can you think quite clearly, or does there seem to be some kind of interference with your thoughts? | No | **0** |
|  |  | Yes | **1** |
| **4** | Do you have unusual experiences that some people have, such as seeing things that others cannot see, having second sight, or being aware of strange presences? | No | **0** |
|  |  | Yes | **1** |
| **5** | Do you ever seem to hear noises or voices when there is nobody about and no ordinary explanation seems possible? | No | **0** |
|  |  | Yes | **1** |
|  |  | Uncertain | **8** |
| **6** | Have you had a feeling that people were too interested in you? | No | **0** |
|  |  | Yes | **1** |
| **7** | Have there been any other odd or unpleasant experiences of any kind recently? | No | **0** |
|  |  | Yes | **1** |
| **8** | Have you had any difficulty with your memory? | None | **0** |
|  |  | Mild difficulty such as “forgetfulness” that might be due to impaired concentration, woes etc. | **1** |
|  |  | Serious memory loss, unlikely to be due to solely to inattention, worry etc. | **2** |
| **Observe the following behaviors of the respondents** | | | |
| **9** | **Slowness**  (Very slow to move. Unusual for age and physical condition. Motor retardation) | No | **1** |
|  |  | Yes | **2** |
| **10** | **Restlessness** | Noticeably restless  (Does not stay still for even 2 secs) | **1** |
|  |  | Please notice how many times she is changing position, standing up, sitting down etc. | **2** |
| **11** | **Odd or inappropriate appearance**  (Clothes, ornaments worn. Is there anything abnormal about her gesture or gait -walking, sitting, running?) | Yes | **1** |
|  |  | No | **2** |
| **12** | **Self neglect**  (clothes inadequate for warmth or protection which is odd and embarrassing) | Yes | **1** |
|  |  | No | **2** |
| **13** | **Anxiety**  (fearful apprehensive look, frightened tone of voice, tremor in voice or hands, autonomic signs) | Yes | **1** |
|  |  | No | **2** |
| **14** | **Depression**  (sad, mournful look, tears, gloomy tone of, deep sighs, voice chokes on distressing topic) | Yes | **1** |
|  |  | No | **2** |
| **15** | **Blunting or flattening of affect**  (Decrease in emotional responsiveness shown in less emotion or facial expression; notice voice tone and movement) | Quantitative decrease compared with expectation | **1** |
|  |  | Severe and uniform flatness of affect | **2** |
| **16** | **Incoherence of speech**  (there is no connection between one part of speech with another, false description, no logical connection between descriptions, sudden irrelevances , abnormal behavior such as: walking in sleep etc. | Yes | **1** |
|  |  | No | **2** |
| **17** | **Magical or illogical (ghost) thinking** | Yes | **1** |
|  |  | No | **2** |
| **18** | **Rate restricted quantity of speech**  (Subject frequently fails to answer, have to repeat the question, restricted to minimum necessary, no extra sentences, no additional comments.) | No | **2** |
|  |  | Yes | **0** |
| **19** | **Poor use of non-verbal communication during interview**  (have to notice lack or under-use of normal non-verbal gestures, facial expressions, change of tone and pitch and loudness, eye contact etc during conversation) | Yes | **1** |
|  |  | No | **0** |
| Score range:0-52 | | Total: | |
